# Spatially-Explicit Estimation of Geographical Representation in Large-Scale Species Distribution Datasets

**DOI:** 10.1371/journal.pone.0085306

**Published:** 2014-01-15

**Authors:** Jesse M. Kalwij, Mark P. Robertson, Argo Ronk, Martin Zobel, Meelis Pärtel

**Affiliations:** 1 Institute of Ecology and Earth Sciences, University of Tartu, Tartu, Estonia; 2 Department of Zoology, University of Johannesburg, Auckland Park, South Africa; 3 Centre for Invasion Biology and Department of Zoology and Entomology, University of Pretoria, Pretoria, South Africa; University of Kent, United Kingdom

## Abstract

Much ecological research relies on existing multispecies distribution datasets. Such datasets, however, can vary considerably in quality, extent, resolution or taxonomic coverage. We provide a framework for a spatially-explicit evaluation of geographical representation within large-scale species distribution datasets, using the comparison of an occurrence atlas with a range atlas dataset as a working example. Specifically, we compared occurrence maps for 3773 taxa from the widely-used Atlas Florae Europaeae (AFE) with digitised range maps for 2049 taxa of the lesser-known Atlas of North European Vascular Plants. We calculated the level of agreement at a 50-km spatial resolution using average latitudinal and longitudinal species range, and area of occupancy. Agreement in species distribution was calculated and mapped using Jaccard similarity index and a reduced major axis (RMA) regression analysis of species richness between the entire atlases (5221 taxa in total) and between co-occurring species (601 taxa). We found no difference in distribution ranges or in the area of occupancy frequency distribution, indicating that atlases were sufficiently overlapping for a valid comparison. The similarity index map showed high levels of agreement for central, western, and northern Europe. The RMA regression confirmed that geographical representation of AFE was low in areas with a sparse data recording history (e.g., Russia, Belarus and the Ukraine). For co-occurring species in south-eastern Europe, however, the Atlas of North European Vascular Plants showed remarkably higher richness estimations. Geographical representation of atlas data can be much more heterogeneous than often assumed. Level of agreement between datasets can be used to evaluate geographical representation within datasets. Merging atlases into a single dataset is worthwhile in spite of methodological differences, and helps to fill gaps in our knowledge of species distribution ranges. Species distribution dataset mergers, such as the one exemplified here, can serve as a baseline towards comprehensive species distribution datasets.

## Introduction

Large-scale species distribution data are widely used in macro-ecology, for example to determine richness patterns in biogeographical studies [1], to estimate species abundances [2], to fill data gaps in monitoring programmes [3] or to assess priority areas for biodiversity conservation [4], [5]. Distribution data obtained from existing atlas datasets or from an open access data publishing framework such as the Global Biodiversity Information Facility database (http://www.gbif.org/), however, vary considerably in data quality, their spatial extent, spatial resolution and taxonomic coverage [6]. Although variability in the geographical representation within datasets is generally acknowledged (see, e.g., [7], [8], [9]), a spatially explicit analysis of where such shortcomings in datasets occur is usually missing [10].

Large-scale comprehensive species distribution datasets are remarkably scarce [3], [4], [11], [12], [13]. This gap is most noteworthy for vascular plants in Europe, given the continent’s long history of botanical research. The Atlas Florae Europaeae (AFE), albeit unfinished, is the only European plant atlas at a fine (50 km) spatial resolution [14], [15], [16], [17]. It has been widely used to describe and analyse European plant patterns (see, e.g., [18], [19], [20], [21], [22]). Indeed, the first thirteen volumes of AFE cover 4123 plant taxa (species or recognised infraspecific taxa) in 3556 occurrence maps, covering 30% of the *∼*13,650 plant taxa in Europe [23]. However, the AFE work progress follows the Englerian taxonomic sequence; starting from pteridophytes, gymnophytes, and angiosperms up to a part of Rosaceae in Vol. 13. A possible sampling bias in this occurrence atlas is, thus, a systematic over-representation of plant species in northern, western and central Europe as volumes covering important Mediterranean families are not published yet [24]. Another problem of AFE is that sampling intensity varies among countries [25], although the extent of this variation is not known.

Comparing and merging large-scale species distribution datasets could help to identify and improve regions that are geographically poorly represented. In the example of the AFE, a comparable dataset would need to contain overlapping species covering the same spatial extent as the AFE as a minimum requirement. This may be achieved with the Atlas of North European Vascular Plants North of the Tropic of Cancer [26] (hereafter referred to as the Hultén & Fries atlas). This atlas consists of continental-level distribution information for *∼*2600 taxa, either in one of the 1936 range maps or in the text description. Furthermore, this range atlas was taxonomically comprehensive and up-to-date for its focus area at the time of publication. Specifically, Hultén & Fries delineated species ranges based on herbarium specimens and the authors’ own observational data in combination with their expert opinion, whereby single occurrences outside such areas were included as single points. False presences in distribution range datasets are a disadvantage, but the degree of this shortcoming depends on the spatial resolution of the data analysis [9]. Therefore, the Hultén & Fries atlas is a suitable comparative dataset to assess the geographical variation in the AFE dataset. A merger of these atlases could be used to fill in gaps in our knowledge of species distribution, while the level of agreement between the two can be used as a spatially explicit assessment of geographical representation within each, and to locate under-sampled areas.

When atlases differ in elementary properties, such as extent of occurrence or area of occupancy, it is essential to determine the extent to which an eventual agreement between the two is valid [6], [27]. The main difference between the AFE and the Hultén & Fries atlas is that the AFE consists of presence data while the latter consists of range maps compiled by the authors (see also Table S1). The Hultén & Fries atlas is thus more likely to contain false presences while the AFE is more likely to include false absences [28]. Nevertheless, since both atlases relied on data provided by local collaborators, we assumed that the primary data sources of these atlases such as local herbaria and publications were largely the same. A subset of the same species from both datasets should thus show similar distribution patterns [9]. Interestingly, the Hultén & Fries atlas used data points from the distribution maps of the first five volumes of the AFE (1972–1980). This would thus provide an additional opportunity to assess the extent to which the Hultén & Fries atlas sampling strategy differed from the AFE, and how this affects the level of agreement between these atlases.

In this study we outline the framework for a spatially explicit analysis of geographical representation in large-scale species distribution datasets, using the AFE and the Hultén & Fries atlas as examples. We compared species diversity patterns of AFE with the Hultén & Fries atlas and for the intersection of species occurring in both atlases by means of a combination of spatially implicit and explicit analyses. We calculated the level of agreement for variables such as species range size and species richness. This enabled us to map the geographical representation within each dataset, and to evaluate the contribution of individual species distribution datasets in a single, merged dataset.

## Materials and Methods

### Study Area

We defined our study area as the intersection between the European continent as delineated by the Atlas Florae Europaeae book series and the Hultén & Fries atlas, roughly between 35–82°N and 31°W–69°E. This means that we included the European parts of the Russian Federation and Turkey, Iceland, and the Svalbard archipelagos, but excluded the Caucasus Mountains region and the archipelagos of Franz Josef Land and Novaya Zemlya. We also excluded the Macaronesian archipelago as this is biogeographically not part of Europe and contains 3106 plant species alone [23]. The climate gradient of our study thus ranged from an Atlantic climate in the west to continental in the east, and from Arctic in the north to Mediterranean in the south of Europe.

### Species Distribution Data

#### Atlas florae europaeae

We obtained digital data of the AFE from the Secretariat of the Committee for Mapping the Flora of Europe. Although two more volumes were published, only the digital data sets of the first 13 volumes were available, covering species distribution data of 4123 taxa under the original taxonomic conception (*personal communication* Alexander Sennikov, Secretary of the Committee for Mapping the Flora of Europe). We excluded records for species that were listed as extinct, probably extinct, or with uncertain identification or locality. This atlas follows the Englerian taxonomic sequence up to and including part of the Rosaceae family (i.e., not a random subset of the current flora in Europe). The spatial resolution of this data set followed the AFE grid system of 2000; a modified Universal Transverse Mercator (UTM) system. This system comprises of squares with a size of *∼*50×50 km with some deviating sizes in the overlapping areas of the UTM zones [17]. In concurrence with the AFE dataset we adopted the same 50-km squares of this AFE grid system (land cover >0%; *n* = 4652) for all analyses unless stated otherwise.

#### Hultén & fries atlas

The Hultén & Fries atlas is an updated and extended version of Hultén’s earlier publications (e.g., [29]). Throughout his work Hultén relied on colleagues from around the world to verify and amend his maps. He also used a standard protocol to collect and file third-party observations, ensuring high data quality and keeping the possibility to verify species identification. Observations of “adventitious, not completely naturalized species, and those escaped from cultivation” were typically excluded [29]. Although Hultén died in 1981, Fries updated and verified all maps to complete the atlas [26]. The Hultén & Fries atlas can thus be considered as comprehensive for its focus area and up to date at the time of publication.

Since the Hultén & Fries atlas was not digitally available, all 1936 maps were scanned at a resolution of 300 dots per inch and georeference into orthomaps. These maps contained (i) point data for species records with isolated, known locations, (ii) polyline data for species with coastal distribution ranges, and (iii) polygon distribution ranges for areas with a common or fairly common occurrence based on the authors’ interpretation of the available data at the time. These data were manually digitised into a geodatabase as point, polyline, and polygon shapefiles for 2605 taxa following the original taxonomic conception. This geodatabase was verified twice to correct any mistakes made during the digitising process: by overlaying shapefiles on the orthomaps and by verifying the attribute table. Digitising of maps and the geostatistical analysis were conducted in ArcGIS 9.3.1 Service Pack 2 (Environmental Science Research Institute, Redlands, CA, USA). We used the vector shapefile of the European continent provided by ArcGIS as a template to digitise the distributions that intersected or overlapped with coastal distributions. Points that were labelled as fossile, extinct, adventive or casual, were excluded from further analysis to ensure that species record status matched that of the AFE. Finally, we determined the presence–absence of each taxon at the same 50-km spatial resolution of the AFE, combining the point, polyline, and polygon shapefiles into a single shapefile. We assigned presences to all squares with entire or partial overlapping attributes, similar to the AFE grid revision approach in which even minimally occupied cells are considered as species present in the entire cell [17].

### Taxonomic Matching

The taxonomic nomenclatures of both the AFE and the Hultén & Fries atlas had undergone significant changes since their publication. We thus carefully verified the taxonomic status of each taxon name using the online database The Plant List (http://www.theplantlist.org/) as our main source of taxonomic information to confirm currently accepted names or to update synonym names. The Plant List was the most comprehensive plant taxonomic database available [30]. This process varied from correcting errors in the original data file (e.g., correcting *Asplenium haussknetchii* to *A. haussknechtii*), to updating old synonyms to currently accepted names (e.g., *Asplenium obovatum* to *A. virillae*) and to merging certain separately mapped taxa (e.g., *Minuartia recurva* sensu stricto and *Minuartia recurva* sensu lato into a single *M. recurva* taxon). Infraspecies taxon level was only retained if listed in The Plant List as an accepted taxon.

### Data Categories

To determine the extent to which the occurrence and the range maps complemented or overlapped each other we considered two analytical categories: the merger of data records from both atlases combined, hereafter referred to as the merged atlas dataset or merger, and an intersection of data records based on plant species that occurred in both atlases, hereafter referred to as the intersection dataset. The AFE collated data such as herbarium specimens and species observations collected by local partners at a national level. Hultén & Fries collated many of the same herbarium specimens, supplemented with their own species observations and with data from existing maps such as Vols 1–5 of the AFE. We therefore split the intersection dataset into two analytical subsets, namely: a dependent data subset, covering records of species that occurred in both the Hultén & Fries atlas and in the first five volumes of the AFE, and an independent data subset, covering records of species that occurred in both the Hultén & Fries atlas and in the other volumes of the AFE (Vols 6–13). All statistical analyses were conducted for each of these four categories unless stated otherwise.

### Statistical Analysis

To quantify the level of agreement in species richness we calculated the slope of the regression between the species richness values of AFE (the occurrence atlas) and the Hultén & Fries (range) atlas using the 50-km squares as observational units (*n* = 4652). Since both datasets were considered as estimated variables we used the reduced major axis (RMA), or model II regression, which is appropriate when both variables are estimated values [31]. We fitted regression models to the number of taxa of the merger, the intersection and the two subsets of dependent and independent data using the R-based lmodel2 package version 1.7-0 [32]. To determine if coastal areas were equally represented in both atlas types, we calculated these RMA models along a range of 0–100% in 10% increments for the minimum proportion of landmass per UTM cell. To illustrate the degree of spatial autocorrelation in each dataset, we calculated Moran’s *I* in 25-km increments for each of the entire datasets and for the residuals of the RMA model, whereby an *I*-value of 0 indicates absence of spatial autocorrelation and a value of 1 indicates complete spatial autocorrelation [33]. Due to the spatial configuration of the study area we calculated distance classes up to 750 km.

To compare species range size distributions we used the area of occupancy and the extent of occurrence. The area of occupancy counts included 50-km squares only, while the extent of occurrence depicts the outer distribution limits [1]. Two extents of occurrence were calculated, namely the longitudinal extent of occurrence (as the geographic difference between the westernmost and easternmost meridians of occupied grid cell centres) and the latitudinal extent of occurrence (between the northernmost and southernmost parallels of occupied cells). The area of occupancy was calculated as the total number of grid cells occupied by each species. To test the statistical difference in range sizes between the two atlases, we used *t*-tests paired by taxon name [31], [34]. These paired analyses were thus only possible for the intersection of species and for the two data subsets. To compare the frequency distribution of species abundance we used the log-transformed areas of occupancy [1]. Histograms were calculated for the complete atlases, for the subset of shared species and for the subset of species that were not shared between atlases.

We used maps to highlight geographical regions of high and low levels of agreement between the two atlases. First we mapped the total number of species for each of the four analytical categories at the 50-km square resolution. We then plotted the residuals of the RMA analysis on a map to point out where deviations between AFE and the Hultén & Fries atlas were strongest. This map served as a spatially explicit representation of which regions displayed poor agreement between datasets. Finally, we calculated the Jaccard index for each cell to determine how the similarity between the atlases was distributed geographically. This Jaccard index emphasizes the difference in species list similarity and is calculated by dividing the intersection of species by the union of species [35].

## Results

### Species Richness

After taxon updating and exclusion of records for extinct species, the AFE data set contained records for 3773 taxa (27% of the original taxa needed some form of nomenclatural updating or editing). The index of the Hultén & Fries atlas listed 4671 taxa, but only 2604 of those were actually mapped. The remaining taxa were either synonyms or merely mentioned in the text accompanying the maps. After updating, the Hultén & Fries atlas contained 2049 mapped taxa (26% of the mapped taxa required nomenclature updating or editing). The merger of the two atlases contained 5221 taxa, of which 601 (12%) were present in both data sets (intersection), representing 29% of the Hultén & Fries atlas and 16% of the AFE atlas data. The species list of the dependent data subset (species maps from the Hultén & Fries atlas that were at least partially based on the AFE atlas) contained 199 taxa. The remaining 402 taxa that were present in both atlases thus belonged to the independent data subset.

Although the Hultén & Fries atlas contained far fewer species, when both atlases were compared in their entirety, the average species richness per 50-km square was 3.34 times higher than in the AFE, although high variation remained (RMA regression analysis: *R* ^2^ = 0.444; Table 1). The same relationship using the intersection of co-occurring species had a slope of 0.960, indicating an almost-perfect relationship between AFE and the Hultén & Fries atlas, although the remaining variation was also high (*R* ^2^ = 0.566). The RMA model fitted to the 199 taxa of the dependent data subset showed that the average richness per 50-km square was lower in the AFE (Hultén = 0.854×AFE+23.044; Table 1), while for the independent data subset this slope was 1.001. Since the confidence intervals of these values did not overlap, we distinguished these data subsets in our subsequent analyses. For all these models the slope values were negatively related to minimum proportion of landmass per UTM cell, indicating that the coastal zone was not equally represented in the two atlas types (Figure S1).

**Table 1:** Results of the reduced major axis (RMA) regression analysis between AFE and the Hultén & Fries atlas for each of the four analytical categories.

Model	*R* ^2^	Slope	95% CI	Intercept	95% CI	*P*-value
Entire atlas	0.444	3.339	3.235–3.450	−19.371	−19.371–16.421	<0.001
Intersection	0.566	0.960	0.936–0.985	42.935	42.935–48.425	<0.001
Independent data subset	0.587	1.001	0.977–1.026	24.107	22.344–25.828	<0.001
Dependent data subset	0.500	0.854	0.830–0.879	23.044	22.015–24.049	<0.001

CI = Confidence interval of the value in the preceding column.

**Figure 1:**
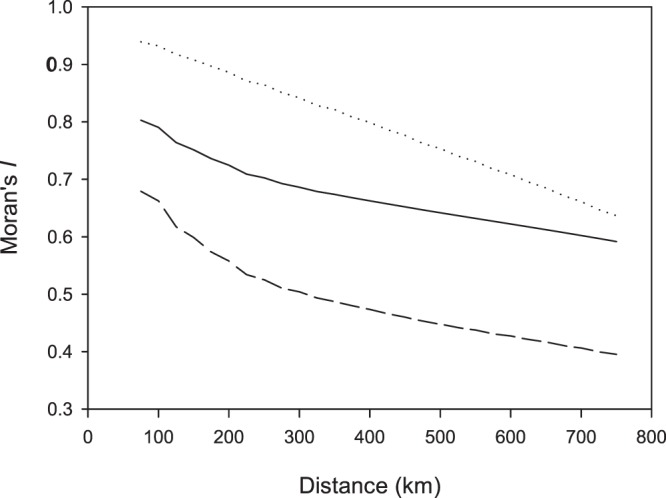
Spatial autocorrelation, expressed as Moran’s *I*, with incrementing distance class for the full datasets of the Hultén & Fries atlas (dotted line) and AFE (solid line), and for the residuals of the reduced major axis model of the two (dashed line).

The Moran’s *I* values showed that species richness was spatially autocorrelated for both datasets with no indication of levelling off at the largest distance class (Fig. 1). The residuals of the RMA model on the full datasets also exhibited spatial autocorrelation, albeit to a lesser extent. These results indicated that the Type I statistical error rate of the RMA model fit prediction was increased and that *R* ^2^ values were deflated [36]. However, no methods currently exist to incorporate spatial autocorrelation into RMA models. Since the *P* values of these models were highly significant (Table 1), we interpreted the slopes and spatial distribution of residuals of these models as such.

### Species Occupancy

With an average of 18.9% of cells occupied for each species, the area of occupancy for the AFE was significantly lower than the 26.1% of the Hultén & Fries atlas (Table 2). In spite of this much lower area of occupancy ratio, neither the latitudinal nor longitudinal extent of occurrence differed significantly between these atlases. The same patterns were found for both data subset categories and were thus not analysed further.

**Table 2:** Results of the *t*-tests of latitudinal and longitudinal ranges and area of occupancy of species.

Category	Variable	*t*	d.f.	*P*-value
Intersection	Latitudinal range	−1.802	600	0.072
	Longitudinal range	0.436	600	0.663
	Area of occupancy	−16.295	600	<0.001
Independent	Latitudinal range	−1.598	401	0.111
	Longitudinal range	1.578	401	0.115
	Area of occupancy	−11.717	401	<0.001
Dependent	Latitudinal range	−0.831	198	0.407
	Longitudinal range	−1.805	198	0.073
	Area of occupancy	−11.881	198	<0.001

All tests were paired by species between the AFE and the Hultén & Fries atlas. A negative *t* value indicates that the AFE value was lower than that of the Hultén & Fries value.

**Figure 2:**
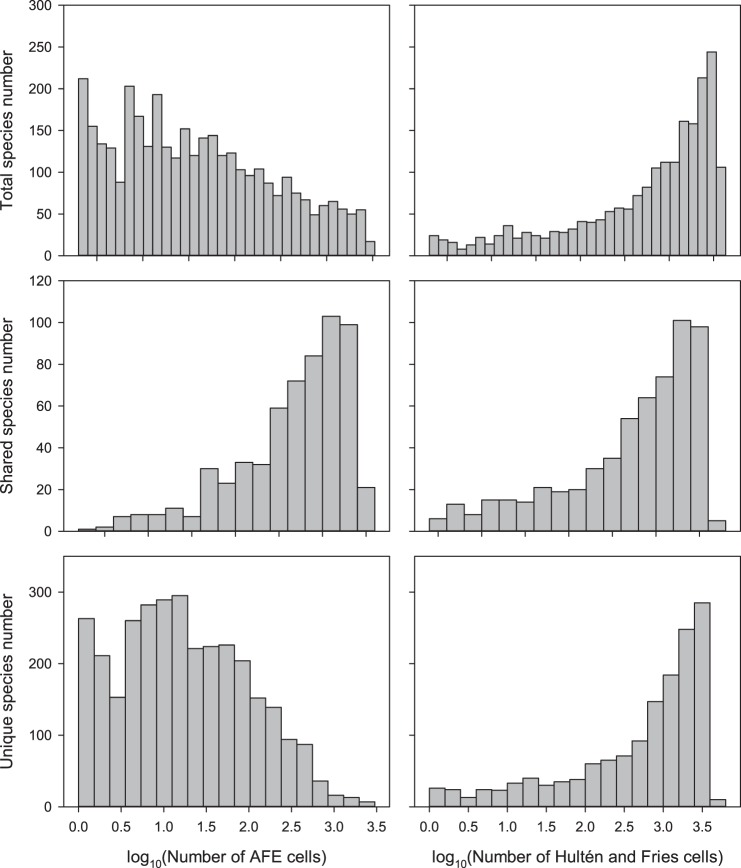
Frequency distributions of the area of occupancy (number of grid cells occupied) and total species number (*n*) for (a) the complete AFE atlas (n = 3773), (b) the complete Hultén & Fries atlas (*n* = 2049), the intersection of species co-occurring in (c) AFE (*n* = 601) or (d) the Hultén & Fries atlas (*n* = 601), species exclusive to (e) AFE (*n* = 3172) or (f) the Hultén & Fries atlas (*n* = 1448).

The area of occupancy histogram for the AFE species showed that this atlas had a moderately right-skewed frequency distribution, indicating that the majority of species had a fairly limited area of occupancy (Fig. 2a). In contrast, the Hultén & Fries atlas showed that most species had a widespread area of occupancy (Fig. 2b). However, the frequency distributions of co-occurring species were very similar (Fig. 2c,d), indicating that the atlases’ sampling methodology did not affect frequency distribution here. Indeed, the area of occupancy distributions of species that were not shared between atlases showed that the AFE had a reasonable number of species with a low to very low area of occupancy (Fig. 2e), whereas that of species exclusive to the Hultén & Fries atlas showed the same left-skewed distribution pattern as the full atlas and the subset of shared species (Fig. 2f).

### Species Richness Distribution

**Figure 3:**
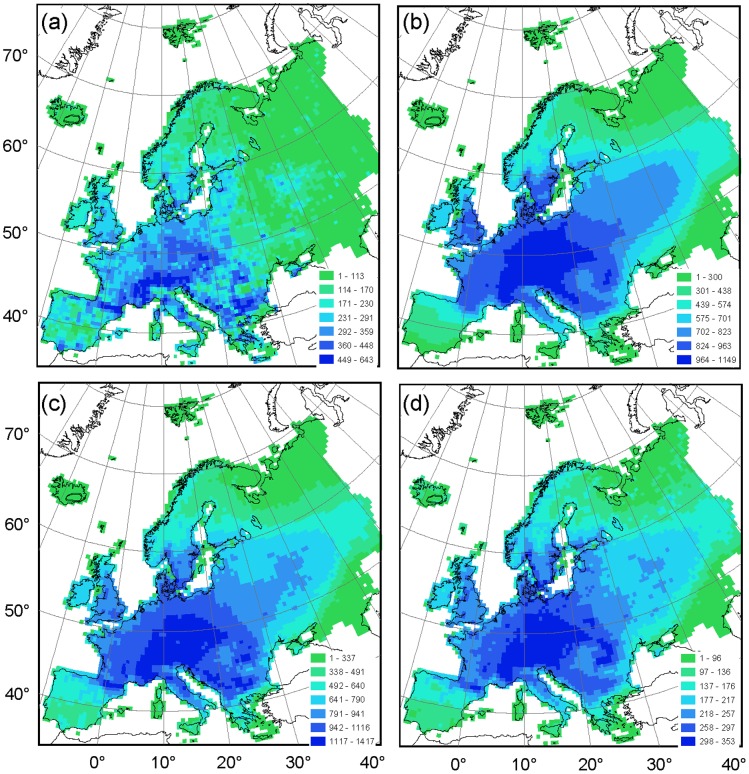
Species richness distribution and the maximum species richness per cell (N_max_) for (a) records of the complete AFE (N_max_ = 643), (b) records of the complete Hultén & Fries atlas (N_max_ = 1149), (c) the merger of the two atlases (N_max_ = 1417) and (d) the intersection of species occurring in both atlases (N_max_ = 353). In each of the panels the relative species richness is illustrated using a seven-category scale legend, where a light grey tone indicates low species richness, and a dark grey tone indicates high species richness. Cells without species records were left empty. Projection: Albers equal-area conic.

Species richness was highest in central Europe for both atlases, in particular for mountainous regions such as the Alps and Pyrenees (Fig. 3a,b; Appendix S1). This pattern was the same for both the merger and intersection of the atlases. However, the AFE species richness pattern was coarse compared to the smooth pattern of the Hultén & Fries atlas. Interestingly, the AFE showed a noticeably higher species richness in the region around Moscow, Russia (55.8°N, 37.6°E), and sharp gradients in species richness for certain political boundaries such as Finland, the Baltic States, and Bulgaria. Such distinct political boundary-associated patterns were not noticeable for the merger or intersection of the two atlases, with the exception of high species richness around Moscow (Fig. 3c,d).

**Figure 4:**
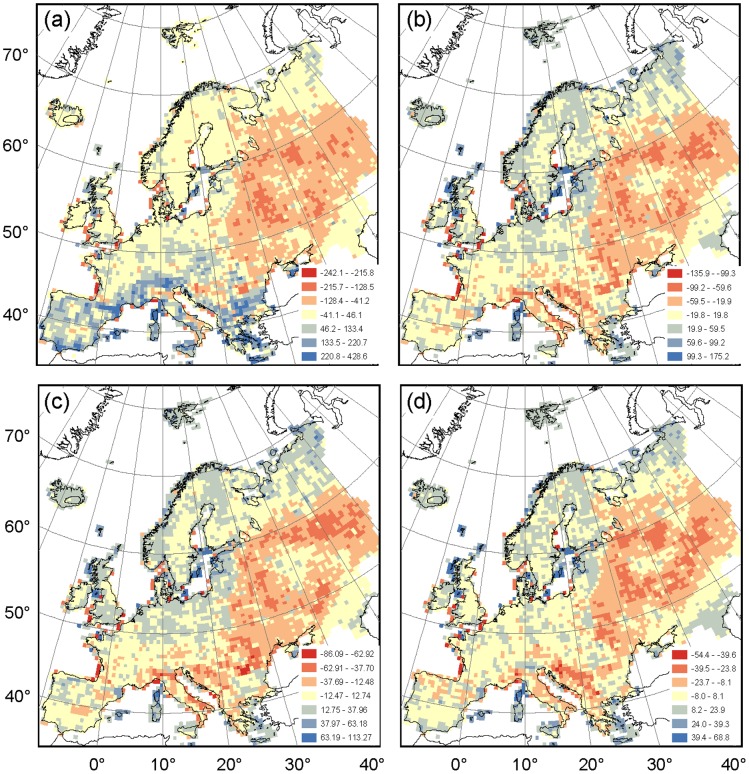
Residual distribution of the reduced major axis (RMA) analysis between species richness of AFE and the Hultén & Fries atlas for (a) the complete atlases, (b) the intersection of species occurring in both atlases, (c) the independent data subset of species mapped in both atlases and (d) the dependent data subset of species. In each of the panels, the deviation from RMA predicted species richness was standardized using a seven-category scale legend, where a red tone intensity illustrated the degree to which the Hultén & Fries atlas richness estimation was higher than the RMA model prediction while the blue tone intensity illustrated level of deviation of the AFE richness estimation.

The RMA residual distribution showed a high level of agreement for Western and central Europe, Poland, the Baltic States and Scandinavia (Fig. 4; Appendix S1). The richness estimations of the entire AFE dataset exceeded RMA model fit in the southern European regions because of the many species that were unique to the AFE (Fig. 4a; blue cells). Since we had no dataset to compare the distribution of these species with, the data quality of these areas in the south was classified as unknown. Furthermore, the AFE richness estimation of co-occurring species was indeed lower than the RMA model prediction for Russia, Belarus and the Ukraine, i.e., the typically *a priori* excluded regions (Fig. 4b–d; red cells). However, AFE richness estimation was also low for most of the south-eastern European countries.

**Figure 5:**
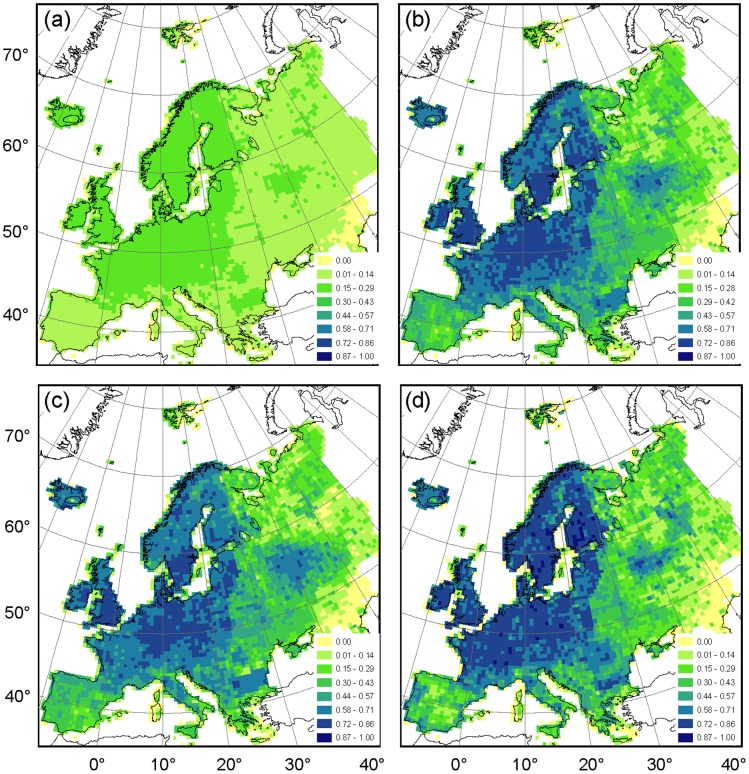
Jaccard similarity index and the maximum index value per cell (J_max_) between (a) the two complete atlases (J_max_ = 0.29), (b) the intersection of species occurring in both atlases (J_max_ = 0.84), (c) the independent data subset of species mapped in both atlases (J_max_ = 0.84) and (d) the dependent data subset of species (bottom-right panel, J_max_ = 1.00). In each of the panels the Jaccard similarity index is illustrated using a seven-category scale legend, where a light grey tone indicates low species list similarity and dark grey tone high species list similarity. Cells with a species similarity index of null were left empty.

The Jaccard similarity index (J) showed a high level of agreement in species lists between the two atlases per 50-km square for Scandinavia, the Baltic States, the British Isles, and north-western Europe (Fig. 5; Appendix S1). This pattern was similar for the complete atlases and for the intersection of the atlases (Fig. 5a,b). There was little difference in level of agreement pattern between the independent and dependent data subsets (Fig. 5c,d). The areas around Moscow and Bulgaria stand out as having remarkably similar species lists for AFE and the Hultén & Fries atlas, similar to the species richness maps.

## Discussion

The sampling intensity of floristic surveys varies considerably among and even within countries [10]. Some have had a long history of detailed botanical mapping (e.g., the Netherlands or Estonia), whereas others (e.g., the former Yugoslavia and Russia) are relatively poorly sampled [23]. Our results confirm such discontinuous gradients in close association with political boundaries (Figs 4 & 5). For this reason, some studies *a priori* exclude areas suspected of being under-sampled, typically European Russia, Belarus and the Ukraine, and work on the assumption that the remainder of the continent is sufficiently and evenly sampled (see, e.g., [21], [37]). However, the level of agreement based on co-occurring species was remarkably low for regions in south-eastern Europe such as Italy and Greece too (Fig. 4b–d). This low geographical representation of AFE for south-eastern European regions is easily overlooked when the entire AFE dataset is considered because of the relatively high number of species in southern Europe with a very limited distribution range. Our comparison with the Hultén & Fries atlas showed that geographical representation of the AFE dataset is much more heterogeneously distributed than often assumed [38], [39]. Therefore, the exclusion of poorly-represented regions must be done based on an evaluation of the spatial distribution of geographical representation, such as the one presented here, rather than on coarse *a priori* assumptions.

### Occurrence *vs* Range Atlas

Variation in atlas project strategy and design, such as sampling intensity or spatial resolution of distribution maps, is often a cause of disagreement between species richness patterns and the products derived from them [7], [40], [41]. For example, a high spatial resolution results in a low occupancy ratio [42]. Indeed, the AFE occurrence atlas had a lower and more scattered species richness pattern than the Hultén & Fries range atlas (Fig. 3a), while the latter was overrepresented in the coastal areas (Figure S1), as would be expected from their respective underlying sampling methodologies [7], [43]. Nevertheless, species range estimations and occupancy frequency histograms of co-occurring species were remarkably similar (Fig. 2c,d; Table 2). Additionally, species exclusive to the occurrence atlas mostly had limited distribution ranges, while most of those species that were exclusive to the range atlas had extensive, pan-European distribution ranges (Fig. 2e,f). This fits the prediction that species of higher latitudes, such as those of a plant atlas of north European species, have a larger distribution range [1]. These results indicate that the mapping protocols themselves did not affect the results of the species occupancy frequency comparison, and that resampling the Hultén & Fries atlas to the 50-km spatial resolution of the AFE was appropriate; two important prerequisites when comparing or merging atlas datasets [9], [44].

Since the production of maps for the Hultén & Fries atlas coincided with the publication of the first five volumes of the AFE, some level of interdependence was inevitable. Indeed, the higher richness values of the Hultén & Fries atlas for the dependent data subset, when compared to the independent data subset, confirmed that the Hultén & Fries atlas drew some data from these AFE volumes (Table 1, Fig. 5). However, subsequent comparisons between these data subsets showed sufficiently similar species diversity metrics to assume that AFE and Hultén & Fries atlas were adequately independent from each other to render a comparison valid [9].

### Merging Atlases

A merger of a single occurrence atlas with a single range atlas may reduce but does not annul the problem of false presences or false absences–to resolve such false presences or absences more datasets would be needed. Also, since the Hultén & Fries atlas is biased towards northern Europe in its species list, it does not provide information on the endemic species of central European mountainous areas or the Mediterranean zone [19], [45]. However, the high level of agreement between atlases for co-occurring species supports the notion that a merger of AFE with the Hultén & Fries range atlas provides supplementary insight in species distribution patterns and an indication of data quality distribution. No such estimation of data quality distribution currently exists for AFE. A merger of these two atlases alone already results in the most comprehensive plant distribution atlas database for Europe to date at a 50-km spatial resolution, containing distribution data for 5221 taxa (∼38% of the estimated 13,650 plant species in Europe [23]). The high species richness radiating from central Europe and in the mountainous regions of Europe that this merger showed is generally in agreement with current predictions [46], [47].

### Prospects and Applications

A common constraint in species distribution datasets is the lack of absence data [6], [28]. For example, AFE contained all available reliable records such as herbarium specimen and published or unpublished observations from partners in each of the European countries [14], [15], [16], [17]. While an attempt was made to fill obvious gaps in species distribution maps, observers did not systematically sample all 50-km squares with equal intensity for presence or absence of species. In addition, the sampling intensity of atlas projects such as the AFE is higher for areas with a higher observer density or a longer history of data collecting [27], [48]. This could explain why species distribution models based on the AFE data are occasionally inaccurate regarding the prediction of the species’ range edges [39]. Although the high reliability of true presence data is advantageous, omission of absence data may lead to inflated false presence rates when, for example, habitat suitability models are calculated [28], or species presence probabilities are estimated [12], [49]. The inclusion of additional data such as the Hultén & Fries atlas or local plant surveys could be used to provide a better estimation of presence and absence probabilities; for example, the Anthos database (http://www.anthos.es) with a spatial resolution finer than the 50-km spatial resolution of the metadata set covering the entire Iberian Peninsula and comprehensive for its many endemic species (see, e.g., [18]), or the open-access online database source initiative GBIF (http://www.gbif.org). However, country-level databases such as the Flora Europaea [50], [51] or the Euro+Med PlantBase (http://www.emplantbase.org/home.html) do not necessarily make a suitable contribution; appending occurrence data with a coarser spatial resolution increases the proportion of false presences (but see [44]).

There is still a shortage of reliable species distribution data covering a large spatial extent at a small-grain resolution [52]. The database and method that we present here can contribute to alleviating this shortage. We identified some areas from which additional data is needed to overcome the current bias in sampling distribution. The database can also be used to test new and existing macroecological hypotheses in a wide range of disciplines and purposes; for example, in the identification of biodiversity hotspots to evaluate conservation priorities [43], to explain species distributions with environmental variables [53], to develop and test new spatial statistical methods [10], or to test the robustness of spatial patterns across scales [36], [52]. In spite of their respective limitations and biases both AFE and the Hultén & Fries atlas can be valuable datasets when studying macroecological or biogeographical patterns, either as a supplement to each or in parallel. Due to its size and extent, we expect that the merger database can explain environmental variables behind biodiversity patterns much better than either atlas alone.

## Conclusions

When comprehensive distribution data are not available, as is most commonly the case, researchers use data of either a taxonomic or a geographic subset and subsequently extrapolate the results to their area of interest. We showed that the level of agreement between two different types of species distribution datasets can be used to evaluate geographical representation with datasets. We also showed that merging atlases into a single dataset is feasible in spite of methodological differences, and can help to fill in gaps in our knowledge of species distribution ranges. Species distribution dataset mergers such as the one exemplified here can serve as a baseline towards comprehensive species distribution datasets.

## Supporting Information

Figure S1 **Main results of the reduced major axis (RMA) models of the Hultén & Fries atlas against the Atlas Florae Europaeae, plotted against the minimum proportion of landmass per UTM cell: (a) R^2^-values, and slopes for the (b) full atlas datasets, (c) intersection of atlas datasets, (d) independent atlas data subset and (e) dependent atlas data subset.**
(DOCX)

Table S1 **Summarizing overview of the basic traits characterizing of the occurrence atlas dataset (AFE) and the range atlas dataset (Hultén & Fries).**
(DOCX)

Appendix S1 (ZIP)
