# Correction: Spatially-Explicit Estimation of Geographical Representation in Large-Scale Species Distribution Datasets

**DOI:** 10.1371/journal.pone.0095065

**Published:** 2014-04-07

**Authors:** The PLOS ONE Staff

## Notice of Republication

This article was republished on March 19, 2014, because of errors in Figures 3-5. The publisher apologizes for these errors.

## Supporting Information

File S1 **Originally published, uncorrected article**.
(PDF)

File S2 **Republished corrected article**.
(PDF)
