# Supplementary material for: Spatially-Explicit Estimation of Geographical Representation in Large-Scale Species Distribution Datasets
Source: PLoS One. 2014 Jan 15;9(1):e85306. doi: 10.1371/journal.pone.0085306 (PMC3893194; doi:10.1371/journal.pone.0085306)
Supplement: Figure S1 — Main results of the reduced major axis (RMA) models of the Hultén & Fries atlas against the Atlas Florae Europaeae, plotted against the minimum proportion of landmass per UTM cell: (a) R2-values, and slopes for the (b) full atlas datasets, (c) intersection of atlas datasets, (d) independent atlas data subset and (e) dependent atlas data subset. (DOCX) [file pone.0085306.s001.docx]

Figure S1. Main results of the reduced major axis (RMA) models of the Hultén & Fries atlas against the Atlas Florae Europaeae, plotted against the minimum proportion of landmass per UTM cell: (a) R^2^-values, and slopes for the (b) full atlas datasets, (c) intersection of atlas datasets, (d) independent atlas data subset and (e) dependent atlas data subset. Although the R^2^-values of the RMA models increased with a higher minimum proportion of landmass per cell, RMA slopes decreased, indicating that a complete or partial exclusion of coastal UTM cells excludes data from the Hultén & Fries atlas and Atlas Florae Europaeae unevenly.
