# Supplementary material for: Spatially-Explicit Estimation of Geographical Representation in Large-Scale Species Distribution Datasets
Source: PLoS One. 2014 Jan 15;9(1):e85306. doi: 10.1371/journal.pone.0085306 (PMC3893194; doi:10.1371/journal.pone.0085306)
Supplement: Table S1 — Summarizing overview of the basic traits characterizing of the occurrence atlas dataset (AFE) and the range atlas dataset (Hultén & Fries). (DOCX) [file pone.0085306.s002.docx]

Table S1. Summarizing overview of the basic traits characterizing of the occurrence atlas dataset (AFE) and the range atlas dataset (Hultén & Fries).

| Trait | AFE | Hultén & Fries |
| --- | --- | --- |
| Dataset type | Occurrence atlas | Range atlas |
| Atlas focus area | Europe | Northern Europe |
| Data distribution range | Restricted to Europe | Comprehensive for the area North of the Tropic of Cancer |
| Data type | Presence in 50-km grid | Points, coastal lines, polygons |
| Observers | Many, varying amongst countries and in time | One observer only (Hultén) |
| Data collection period | 1965–2004^1^ | 1927–1985^2^ |
| Bias concern | Taxonomically biased | Geographically biased |
| Number of taxa (after nomenclatorial updating) | 3773 | 2049 |
| Species overlap with other dataset (%) | 15.9% | 29.3% |

^1^ We used the founding year of the Committee for Mapping the Flora of Europe as indicated in the introduction of Volume 1 of the AFE as starting date of data collecting.

^2^ The Hultén & Fries atlas includes all of Hultén’s earlier work. Therefore we assumed here that his collection period commenced with the publication of Hultén’s first flora series: Hultén, E. 1927–1930. *Flora of Kamtchatka and the adjacent islands*. Vols 1–4, Almqvist & Wiksell, Stockholm, Sweden.
